# Participation in cost-offset community-supported agriculture by low-income households in the USA is associated with community characteristics and operational practices

**DOI:** 10.1017/S1368980022000908

**Published:** 2022-08

**Authors:** Karla L Hanson, Lynn Xu, Grace A Marshall, Marilyn Sitaker, Stephanie B Jilcott Pitts, Jane Kolodinsky, April Bennett, Salem Carriker, Diane Smith, Alice S Ammerman, Rebecca A Seguin-Fowler

**Affiliations:** 1 Department of Public and Ecosystem Health, Shurman Hall, Cornell University, Ithaca, NY 14853, USA; 2 The Evergreen State College, Olympia, WA, USA; 3 Brody School of Medicine, East Carolina University, Greenville, NC, USA; 4 Center for Rural Studies, University of Vermont, Burlington, VT, USA; 5 Cornell Cooperative Extension of Jefferson County, Watertown, NY, USA; 6 University of South Carolina, School of Medicine, Columbia, SC, USA; 7 Washington State University, Extension of Skagit County, Burlington, WA, USA; 8 Center for Health Promotion and Disease Prevention, University of North Carolina at Chapel Hill, Chapel Hill, NC, USA; 9 Texas A&M AgriLife Research, College Station, TX, USA

**Keywords:** Community-supported agriculture, Community characteristics, Operational practices

## Abstract

**Objective:**

Subsidised or cost-offset community-supported agriculture (CO-CSA) connects farms directly to low-income households and can improve fruit and vegetable intake. This analysis identifies factors associated with participation in CO-CSA.

**Design:**

Farm Fresh Foods for Healthy Kids (F3HK) provided a half-price, summer CO-CSA plus healthy eating classes to low-income households with children. Community characteristics (population, socio-demographics and health statistics) and CO-CSA operational practices (share sizes, pick up sites, payment options and produce selection) are described and associations with participation levels are examined.

**Setting:**

Ten communities in New York (NY), North Carolina (NC), Vermont and Washington states in USA.

**Participants:**

Caregiver–child dyads enrolled in spring 2016 or 2017.

**Results:**

Residents of micropolitan communities had more education and less poverty than in small towns. The one rural location (NC2) had the fewest college graduates (10 %) and most poverty (23 %) and poor health statistics. Most F3HK participants were white, except in NC where 45·2 % were African American. CO-CSA participation varied significantly across communities from 33 % (NC2) to 89 % (NY1) of weeks picked up. Most CO-CSA farms offered multiple share sizes (69·2 %) and participation was higher than when not offered (76·8 % *v*. 57·7 % of weeks); whereas 53·8 % offered a community pick up location, and participation in these communities was lower than elsewhere (64·7 % *v*. 78·2 % of weeks).

**Conclusion:**

CO-CSA programmes should consider offering a choice of share sizes and innovate to address potential barriers such as rural location and limited education and income among residents. Future research is needed to better understand barriers to participation, particularly among participants utilising community pick up locations.

Fruit and vegetable (FV) consumption is associated with reduced risk for chronic disease and other positive health outcomes^(1,2)^. However, most US populations do not consume recommended amounts of FV^(3)^. One approach to improving FV intake is community-supported agriculture (CSA), which directly connects local farms to consumers by allowing community members to purchase a share of a farm’s anticipated harvest^(4)^. Because a lump sum payment is typically required before the growing season begins, CSA may be more accessible to households with higher incomes^(5–9)^. However, the economic and health benefits of CSA participation for low-income households have been documented^(10,11)^. To broaden access to CSA, some programmes offer a subsidy, or cost-offset (CO-CSA), to low-income participants who are at greater risk of food insecurity and poor nutritional intake^(12,13)^. Understanding the community characteristics and operational practices that support participation in CO-CSA would provide useful implementation information to farms and other CO-CSA programme operators.

To our knowledge, twelve prior studies have examined CO-CSA effectiveness in improving FV access and intake, as well as household food security^(14–25)^ and most, but not all, report beneficial changes in outcomes. For example, a pilot study reported that all participants consumed a greater variety of vegetables, learned new methods for cooking and preparing vegetables and liked new vegetables after participating in the CO-CSA^(20)^. Descriptive studies suggest that CO-CSA participation is associated with improved FV access^(12,20)^ and intake^(18,25)^, and that participants bought more fresh produce, tried new recipes and cooked with more vegetables after joining a CO-CSA^(16)^. Longitudinal studies of CO-CSA more often reported increases in FV intake^(15,17,19,21,23)^ than no changes^(14,20,22)^. Findings from one randomised controlled trial supported the effectiveness of CO-CSA relative to unconditional cash transfer in terms of improved diet quality and reduced food insecurity^(17)^. Taken together, these studies suggest that CO-CSA participation can have positive effects on FV access, dietary intake and related behaviours.

These studies examined CO-CSA implemented in different community contexts and enrolled participants who had varying characteristics. Seven studies operated in urban areas^(14,15,19–22,24)^, and five in predominately rural and micropolitan communities^(16–18,23,25)^. Almost half enrolled participants from communities that were predominately African American and/or Hispanic^(14,15,19,22,24)^. Most CO-CSA participants were women (71–92 %) of all ages, but sometimes were limited to caregivers of children^(16,18,19,25)^, Head Start staff^(19)^ or focussed on older adults^(21,24).^

The CO-CSA programmes examined in prior studies also varied in operational practices. Five studies examined CO-CSA programmes that were free^(15,16,21,22,24)^, but others required either a contribution based on a fixed percentage of share price – 45 %^(14)^, 50 %^(18,23,25)^ or 67 %^(19)^ – or a sliding scale (43–75 %) based on ability to pay^(17,20)^. CO-CSA programmes varied in the options offered to participants, with multiple farm partners resulting in multiple share sizes^(14,16)^, multiple pick up locations^(16,23)^ and/or delivery^(16,21,24)^, or these options were offered at some farms but not others^(18,25)^. Four of five CO-CSA programmes that partnered with just one farm offered only one option for share size and pick up location^(15,19,20,22)^. Three studies assessed CO-CSA programmes that operated with market-style selection of produce items^(17,20,24)^, or in which item selection was available at some farms^(18,25)^. Of the seven studies of CO-CSA programmes that required payment, five accepted Supplemental Nutrition Assistance Programme (SNAP) benefits as payment^(14,17,19,20,23)^ and two accepted SNAP at some farms^(18,25)^.

Farm Fresh Foods for Healthy Kids (F3HK), the focus of this manuscript, was a summer growing season CO-CSA programme (mean = 21 weeks, interquartile range 19–23)^(26)^, provided at 50 % cost-offset (half-price)^(27)^. The CO-CSA was supported by nine CSA-tailored, in-person nutrition education lessons that featured seasonal produce items via food tastings, demonstrations, hands-on cooking activities, handouts and recipes, and by providing participants with two to four larger cooking tools (e.g. food processor and crockpot)^(27)^. The F3HK intervention was implemented in the context of a randomised controlled trial with 1:1 random assignment of child–caregiver dyads to intervention and control groups^(27)^. F3HK reported overall improvements relative to control in caregiver nutrition attitudes, self-efficacy, FV intake, skin carotenoids, preparation of FV as snacks for children and household food security^(28)^. However, F3HK was implemented in small and micropolitan communities across four states, each with a unique context for implementation. Although F3HK required farms to receive weekly payments from participants and to accept SNAP as payment^(27)^, all other operational practices were decided by each partner farm.

The focus of this manuscript is to explore the context of F3HK implementation with respect to community and participant characteristics and CO-CSA operational practices and to examine associations with participation levels. First, we describe the population characteristics of communities in which the F3HK intervention was implemented. Second, we explore differences in F3HK participant characteristics across communities and states and contrast participant and population characteristics. Third, we describe CO-CSA operational practices at F3HK partner farms and explore differences across communities and states. Fourth, we examine associations between community characteristics, participant characteristics, CO-CSA operational practices and participation levels (i.e. percent of CO-CSA weeks picked up, percent of CO-CSA nutrition lessons attended). These analyses inform recommendations for the development and implementation of future CO-CSA programmes in varied settings.

## Methods

### Setting and participants

Target locations for F3HK implementation were small and micropolitan (≤ 50 000 population) communities in New York (NY), North Carolina (NC), Vermont (VT) and Washington (WA) in the USA. Each community also had to be served by a partner farm experienced in CSA and a nutrition educator (through cooperative extension in three of four states). F3HK was implemented in a total of twelve communities. In most communities, one farm provided the CO-CSA. However, in VT1 and WA1 participants chose between two different farms, and in all communities in NC, participants were selected from among the same farms. Throughout this article, ‘community’ refers to the location and ‘farm’ refers to the CO-CSA provider.

**Fig. 1:**
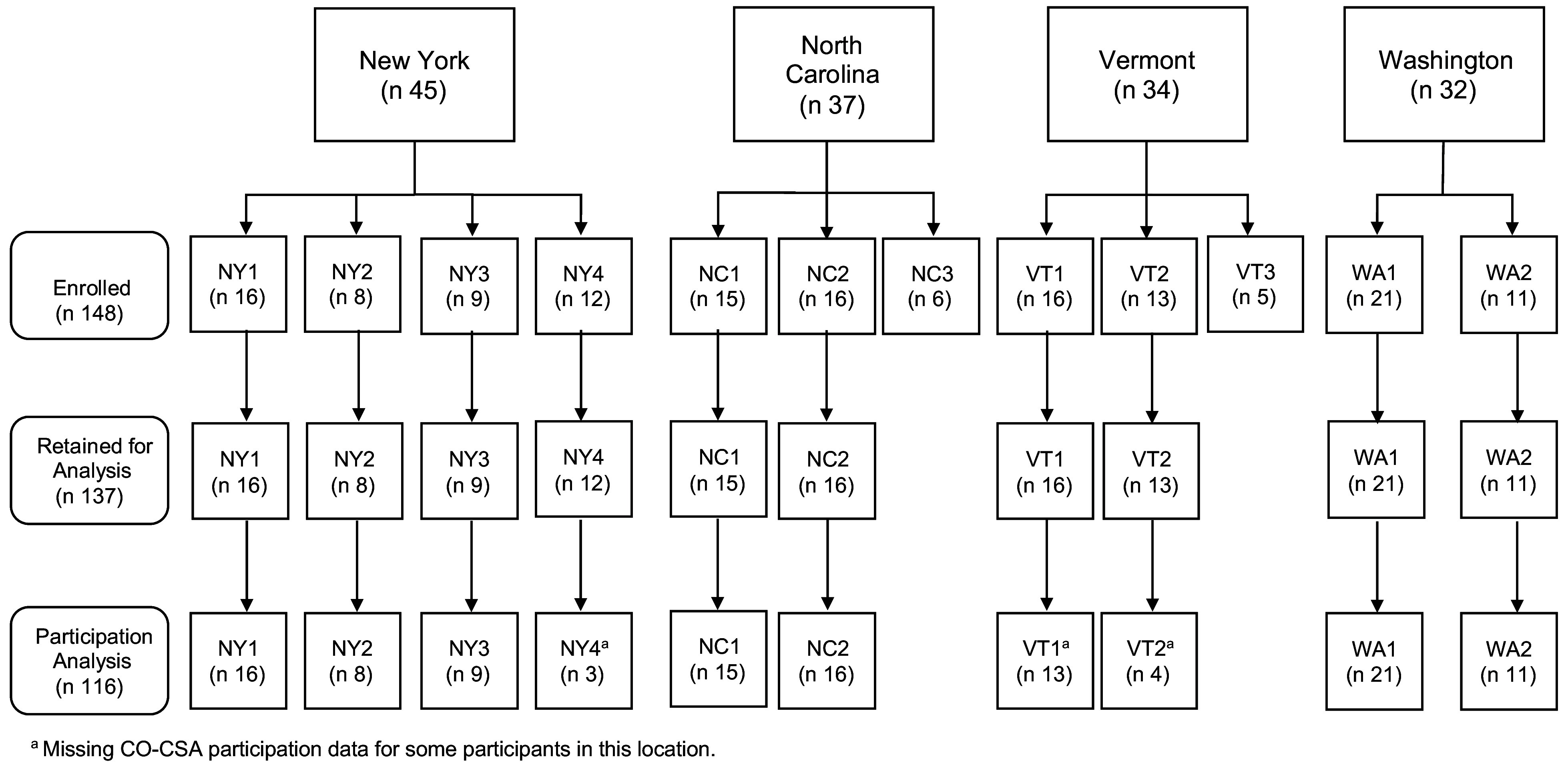
F3HK implementation states, communities, and participants included in analyses

In spring 2016 and 2017, F3HK enrolled English-speaking adult caregivers of a child 2–12 years of age who had self-reported household income ≤185 % of the Federal Poverty Level and had not participated in CSA for the past 3 years. Participants agreed to use SNAP benefits or money to pay for the CO-CSA share weekly and to attend nine CSA-tailored nutrition education classes^(27)^. A total of 685 caregivers were screened for eligibility, 542 (79·1 %) were eligible and 305 of those enrolled (56·3 %), of which 148 were assigned to the intervention group^(28)^. Two communities (one each in NC and VT) were excluded from this analysis due to low F3HK enrolment (≤7 participants, >1 sd below mean sample size of 12), and the remaining 137 intervention participants in 10 communities are the focus of this analysis. Figure 1 depicts the states, communities and participants included in these analyses.

### Measures

Six community characteristics and seven county health statistics were obtained from publicly available data for the approximate time period of F3HK implementation. Community characteristics included population^(29)^, percent of adult population with at least a bachelor’s degree^(30)^, percent of households in poverty, percent of children in poverty, race and ethnicity^(31)^. We subsequently categorised towns as small (population ≤15 000) or micropolitan (population >15 000). Rural-urban continuum (RUCA 2010) codes also were recorded^(32)^. County health statistics included percent of persons food insecure^(33)^, and percent of adult residents with diabetes, high cholesterol, high blood pressure, overweight, obesity^(34)^ and cancer^(35)^.

Seven CO-CSA operational practices were extracted from partnership agreements, farm websites and personal communications and dichotomised for analysis: location (outside or within the participant community), length of summer CSA (≤21 weeks or longer), number of pick up sites offered (one or multiple), number of share sizes offered (one or multiple), payment options (credit or debit cards accepted or not) and whether market-style self-selection of produce items or ‘u-pick’ options were offered or not. U-pick allows consumers to harvest larger quantities of certain produce items themselves. Participant selection of type of pick up site (farm, community location or farmer’s market/farm stand) and type of share size (small (≤8 produce items/week) or large (>8 items)) were recorded as part of a baseline survey.

Participant characteristics were recorded during eligibility screening or online baseline survey. Caregiver characteristics included caregiver sex, general health, marital status, education level, employment status, race and ethnicity. Child’s age, sex and general health also were reported by the caregiver. Household characteristics included number of adults and number of children in household, annual household income and receipt of food assistance benefits through the Special Supplemental Nutrition Program for Women, Infants and Children or SNAP in the past month^(27)^. Few participant characteristics were missing (maximum 0·7 %), and missing items are noted on tables.

Participation levels were assessed as: (1) percent of CO-CSA weeks picked up as recorded on logs maintained by partner farms and (2) percent of CSA lessons attended as recorded on lesson sign-in sheets^(26)^.

### Analyses

Community characteristics and county health statistics were rounded before reporting to maintain the confidentiality of F3HK communities. Descriptive statistics were used to summarise participant characteristics, CO-CSA options selected by participants and participation levels by community and state. Pearson’s chi-square, Fisher’s exact tests and one-way and Welch’s ANOVA with Bonferroni correction were used to test for differences across communities and states. One-way ANOVA was used to test differences in participation levels across community size, participant characteristics and CO-CSA operational characteristics. Two farms (NY4 and VT2) did not provide reliable CO-CSA pick up data, and the twenty-five participants at these locations were excluded from analyses of weeks picked up. All analyses were performed using SPSS version 26 (IBM Corp.). Results are reported at a 95 % confidence level.

## Results

### Community and participants characteristics

Community characteristics varied across the 10 F3HK communities included in this analysis (Table 1). Populations ranged from 7000 to 60 000 (just above the upper bound typically used when classified as micropolitan). The largest communities (60 000, 50 000 and 40 000 population) also had the most education (77 %, 44 % and 53 % held a college degree, respectively). The two communities in NC were distinct from one another: NC1 had the highest percentage of adults with at least a bachelor’s degree (77 %), whereas NC2 had the lowest rate of college graduates (10 %) and the highest rate of poverty (23 % of households). The smallest community (NY4) had the lowest rate of poverty (4 % of households).

**Table 1:** Characteristics of communities where F3HK was implemented

Community characteristics	New York				North Carolina		Vermont		Washington	
	NY 1	NY 2	NY 3	NY 4	NC 1	NC 2	VT 1	VT 2	WA 1	WA 2
	%	%	%	%	%	%	%	%	%	%
Population*	25 000	20 000	10 000	7000	60 000	10 000	40 000	15 000	50 000	9000
College educated‡	21	22	39	14	77	10	53	28	44	19
Households in poverty†	20	10	11	4	4	23	12	12	11	12
Children in poverty†	43	20	22	6	5	37	26	26	20	31
Race†										
American Indian/Alaskan Native	1	0	0	0	0	0	0	0	1	0
Asian	1	1	2	0	13	2	6	2	7	4
Black/African American	8	8	2	1	10	22	5	1	3	1
Native Hawaiian/Pacific Islander	0	0	0	0	0	0	0	0	0	1
White	84	86	93	97	73	61	85	96	83	75
None of the above	1	1	1	0	2	9	0	0	1	17
Hispanic†	8	4	2	0	7	43	3	1	8	30
County health statistics										
Food insecurity§	13	12	12	12	13	11	12	12	12	11
Diabetes‖	8	9	10	9	9	12	7	8	8	9
High cholesterol‖	28	32	32	32	28	36	24	29	29	31
High blood pressure‖	27	31	31	31	29	38	25	30	29	31
At least overweight‖	61	62	62	62	62	68	59	62	63	65
Obese‖	27	27	26	27	29	32	26	27	29	30
Cancer¶ per 100 000 pop.	533	541	478	541	442	438	459	476	475	469

Sources:

* US Census Bureau. Total population, 2011–2015 American community survey (ACS) 5-year estimates: US Census Bureau; 2015 (Available from: https://data.census.gov/cedsci/).

† US Census Bureau. Population density, household income, families in poverty, people in poverty, race, ethnicity, 2014–2018 American community survey (ACS) 5-year estimates: US Census Bureau; 2018 (Available from: https://data.census.gov/cedsci/).

‡ US Census Bureau. Educational Attainment, 2015–2019 American Community Survey (ACS) 5-Year Estimates: US Census Bureau; 2019 (Available from: https://data.census.gov/cedsci/).

§ Feeding America 2017 (Available from: http://map.feedingamerica.org/).

‖ Centers for disease control and prevention, 2017 BRFSS survey 2017 (Available from: https://www.cdc.gov/brfss/annual_data/annual_2017.html).

¶ Centers for Disease Control and Prevention and National Cancer Institute. Cancer Incidence, 2013–2017: Centers for Disease Control and Prevention and National Cancer Institute; 2017 (Available from: https://gis.cdc.gov/Cancer/USCS/DataViz.html).

Residents of most communities were predominantly white and non-Hispanic, except in NC2 where 22 % of residents were Black or African American and 43 % were Hispanic and in WA2 where 30 % of residents were Hispanic. NC2 also had the highest rates of diabetes (12 %), high cholesterol (36 %), high blood pressure (38 %), overweight (68 %) and obesity (32 %) but also the lowest rate of cancer (438 per 100 000). All NY locations had high cancer rates (478–541 per 100 000). Across all communities, 11–13 % of households were food insecure.

Some F3HK participant characteristics varied significantly across communities and states (Table 2) and mirrored some of the overall community differences described above. For example, participant race and household income differed significantly by state; NC had the most Black or African American participants (45·2 %). Population data showed that both NC2 and WA2 had high percentages of Hispanic residents (43 % and 30 %, repectively), and both recruited relatively low percentages of Hispanic participants (4 participants or 25 %, and 1 or 9 %, respectively). NC was also the state with the lowest percentage of participants with household income ≥$25 000 (29·0 %). Household income also varied significantly across communities, with NC2 having a notably low percentage of participants with income ≥$25 000 (12·5 %). Three respondents in NC and three in VT (10 % each) had a child in fair or poor health whereas none did in either NY or WA.

**Table 2:** F3HK participant characteristics by community and state

Household characteristics	New York					North Carolina			Vermont			Washington			Sig. by state	Sig. by community
	NY1 (*n* 16)	NY2 (*n* 8)	NY3 (*n* 9)	NY4 (*n* 12)	NY Total	NC1 (*n* 15)	NC2 (*n* 16)	NC Total	VT1 (*n* 16)	VT2 (*n* 13)	VT Total	WA1 (*n* 21)	WA2 (*n* 11)	WA Total		
	#	#	#	#	%	#	#	%	#	#	%	#	#	%		
≥2 Adults	14	5	7	8	75·6	12	8	64·5	11	8	65·5	15	6	65·6		
≥3 Children	7	3	3	7	44·4	4	4	25·8	3	4	24·1	10	3	40·6		
Income ≥$25 000	12	5	7	6	66·7	7	2	29·0	10	4	48·3	8	6	43·8	*	*
Supplemental Nutrition Assistance Programme in past mo.†	5	5	3	5	40·0	9	10	61·3	5	8	46·4	12	5	53·1		
Caregiver characteristics																
Race																
White	14	5	9	10	84·4	5	9	45·2	13	12	86·2	17	10	84·4	*	
Black/African American	0	3	0	1	8·9	8	6	45·2	2	0	6·9	1	0	3·1		
Other/Multiple	2	0	0	1	6·7	2	1	9·7	1	1	6·9	3	1	12·5		
Hispanic	1	0	0	0	2·2	1	4	16·1	0	0	0·0	2	1	9·4		
≥College degree	13	5	5	5	62·2	8	4	38·7	11	6	58·6	9	6	46·9		
Employed	7	3	5	4	42·2	10	7	54·8	6	8	48·3	7	4	34·4		
Married	12	3	5	6	57·8	4	5	29·0	11	5	55·2	8	3	34·4		
Fair/poor health	0	2	3	3	17·8	5	4	29·0	4	1	17·2	3	2	15·6		
Child characteristics																
≤5 years of age	9	2	3	3	37·8	5	7	38·7	12	6	62·1	11	7	56·3		
Fair/poor health	0	0	0	0	0·0	1	2	9·7	2	1	10·3	0	0	0·0	*	

Differences across communities and states were tested using Pearson’s chi-square or Fisher’s exact tests all with Bonferroni correction.

* Indicates difference at > 95 % confidence.

† One observation missing from VT1 for this measure.

### CO-CSA operational practices

Four farms (30·8 %) were located within the participant community, two of which were within VT1 (Table 3). Six farms (46·2 %) operated summer CSA shares lasting 22+ weeks, all of which were in NY and NC. Most farms offered multiple share sizes (9 or 69·2 %) or a community pick up location (7 or 53·8 %), and many offered market-style produce selection (6 or 46·2 %), payment by credit/debit card (6 or 46·2 %) and multiple pick up locations (4 or 30·8 %). When participants were offered a choice of share sizes (*n* 85), the majority (63·5 %) selected a larger share (>8 produce items/week); and, when offered multiple locations for CO-CSA pick up (*n* 41), most (61·0 %) chose a community pick up site (data not shown).

**Table 3:** Cost-offset community-supported agriculture (CO-CSA) operational practices by community

		New York				North Carolina*	Vermont		Washington	
	Total	NY1	NY2	NY3	NY4	NC1 & 2	VT1	VT2	WA1	WA2
	#	#	#	#	#	#	#	#	#	#
# of Farms	13	1	1	1	1	3	2	1	1	2
	%									
Farm located within community	30·8	0	1	0	1	0	2	0	0	0
Longer CSA season (22+ weeks)	46·2	1	1	1	0	3	0	0	0	0
Farm offered:										
Multiple share sizes	69·2	1	1	1	1	0	2	1	1	1
Multiple pick up sites	30·8	0	0	0	0	2	0	1	0	1
A community pick up site	53·8	0	0	0	0	3	1	0	1	2
Payment by credit/debit card	46·2	1	1	0	1	3	0	0	0	0
Market-style produce selection	46·2	0	1	1	0	1	2	1	0	0
U-pick produce items	15·4	0	1	0	0	0	1	0	0	0

* Partner farms shared enrolment of participants in NC1 and NC2.

Farms in VT and WA offered the most flexibility in share size and pick up options, but pick up locations often shifted over time. For example, in WA1 pick up initially occurred at a public housing complex and shifted to a downtown non-profit serving families with low incomes for the second year. In WA2, one farm initially held pick up at a downtown visitors’ information center and the other at the community programmes office of the local hospital, but both farms shifted for the second year (the first to the local hospital and the second away from the hospital and to their own farm stand). Other states offered fewer options. Almost all NY farms offered a choice in share size, but no NY farm initially offered a community pick up site or a choice of pick up locations. In NY2 however, pick up locations also shifted over time to include a community location at which participants could pick up a missed share and later the farm used that location exclusively when the farmers’ market closed for the season. In NC, on the other hand, no farm offered a choice of share sizes, but two of three farms offered a choice of pick up locations and all three offered a community pick up site.

### Participation level

Participation varied significantly across communities: participants picked up the CO-CSA share 33–89 % of weeks and attended 15–58 % of the education lessons (Table 4). In most communities, participants picked up their CSA share three-quarters of the weeks or more often. CO-CSA participation was lowest in NC2 (33·4 %) and highest in NY1 (89·2 %). Attendance at nutrition education classes also differed significantly across communities, with the lowest attendance in NC2 (15·3 %) and highest attendance in NC1 (57·8 %). CO-CSA pick up and class attendance behaviours were aligned in NC2 (low participation) and in NC1 and VT1 (high participation). In five communities, however, percentages for class attendance were 40–60 percentage points below CO-CSA pick up participation level (NY1–3 and WA1–2). There were no significant differences in participation level across states.

**Table 4:** Mean participation level by community and state

Mean participation level	*n*	Total	New York					North Carolina			Vermont			Washington			Sig. by state	Sig. by community
			NY1	NY2	NY3	NY4	NY Total	NC1	NC2	NC Total	VT1	VT2	VT Total	WA1	WA2	WA Total		
% CO-CSA weeks picked up	116	70·9	89·2	74·9	73·7	44·9	78·5	77·9	33·4	55·0	76·6	81·3	77·7	80·6	61·8	74·1		*
% Education lessons attended	137	31·1	31·9	27·8	27·2	31·5	30·1	57·8	15·3	35·8	47·9	30·8	40·2	20·1	19·2	19·8		*

CO-CSA, cost-offset community-supported agriculture.

* Indicates difference > 95 % confidence.

Differences in means were tested using one-way and Welch’s ANOVA with Bonferroni correction.

Compared to households in small towns, households in micropolitan communities had significantly higher participation in the CO-CSA (80·6 *v*. 54·4 % of weeks) and education classes (36·7 *v*. 24·2 % of lessons; Table 5). CO-CSA participation also was higher among households that included at least two adults (78·5 *v*. 54·5 % of weeks), included a young child (80·3 *v*. 62·6 %), and those with incomes at least $25 000/year (84·5 *v*. 57·1 %). Caregivers with a college education (82·6 *v*. 57·8 %) and those who were married (84·5 *v*. 60·6 %) also picked up CO-CSA shares a greater percentage of weeks than their counterparts. Married caregivers also attended significantly more education lessons than unmarried caregivers (38·2 *v*. 25·3 % of lessons), which was the only variation in lesson attendance across any participant characteristic. CO-CSA participation also was notably lower in the six households that included a child in fair or poor health (38·9 *v*. 72·3 % of weeks).

**Table 5:** Mean participation level by community size, participant characteristics, and cost-offset community-supported agriculture (CO-CSA) operational practices

	% CO-CSA weeks picked-up (*n* 116)			% Education lessons attended (*n* 137)		
	No	Yes		No	Yes	
Community size >15 000 population	54·5	80·6	*	24·2	36·7	*
Household characteristics						
≥ 2 Adults in household	54·5	78·5	*	25·1	33·9	
≥ 3 Children in household	68·0	76·1		33·7	26·4	
Household income ≥$25 000	57·1	84·6	*	27·5	35·0	
Household received Supplemental Nutrition Assistance Programme †	75·8	65·8		30·0	32·3	
Caregiver characteristics						
White	60·7	74·9		31·3	31·1	
Hispanic	72·3	53·9		31·4	27·2	
Married	60·6	84·5	*	25·3	38·2	*
College educated	57·8	82·6	*	27·7	34·3	
Employed	70·2	71·7		31·3	31·0	
Fair to poor health	73·9	59·2		30·5	33·7	
Child characteristics						
≤ 5 years of age	62·6	80·3	*	32·3	29·9	
Fair to poor health	72·3	38·9	*	31·2	29·6	
CO-CSA operational practices						
Farm located within community	70·6	72·0		28·7	38·0	
Longer CSA season (22+ weeks)	75·4	66·7		28·6	31·4	
Offered:						
Multiple share sizes	57·7	76·8	*	34·0	30·1	
Multiple pick up sites	74·5	61·4		29·6	34·7	
A community pick up site	78·2	64·7	*	34·6	27·4	
Payment by credit/debit card	75·9	65·4		28·4	34·2	
Market-style produce selection	71·4	69·8		29·9	33·1	
U-pick options	69·7	75·9		28·8	43·9	

Differences in means were tested using *t*-tests with Bonferonni correction.

* Indicates difference at > 95 % confidence.

† One observation missing for this measure in both analyses.

When considering CO-CSA operational characteristics, CO-CSA participation level was higher when farms offered multiple share sizes (76·8 *v*. 57·7 % of weeks) and lower when farms offered a community pick up site (64·7 *v*. 78·2 % of weeks; Table 5). Farm location in the community, longer summer CSA length, multiple pick up sites, payment by credit/debit card, market-style selection of produce and u-pick options all were not associated with participation level. Attendance at education lessons did not differ by any CO-CSA operational practices.

## Discussion

This study reported significant differences in CO-CSA and education participation levels across communities but did not vary across the four states in which F3HK was implemented. This provides novel evidence that suggests that the local setting may matter more in supporting participation than does the state context. Further, we identified four inter-related characteristics (micropolitan location, education, income and spouse or other adult in the household) and two distinct operational practices (offering multiple share sizes and community pick up) that were associated with CO-CSA participation level and should be considered when adapting and implementing CO-CSA for rural and micropolitan communities. Five prior studies of CO-CSA took place in predominately rural and micropolitan areas^(16–18,23,25)^, but these studies provided few details about their local contexts. A few prior CO-CSA studies documented widely varying participation levels^(14,17,19,22)^, but only one was in a micropolitan community^(17)^, none operated CO-CSA in multiple states and no study examined how community characteristics, participant characteristics or programme operations were associated with participation levels.

### Characteristics that may support CO-CSA participation

Participants in micropolitan communities had higher participation levels in both the CO-CSA and the CSA-tailored education classes. To our knowledge, no prior research contrasted CO-CSA participation levels in rural and micropolitan communities. Our data provide novel evidence that residents of micropolitan areas may be better able to participate in CO-CSA than their rural counterparts. We noted some population characteristics that differed between micropolitan and rural communities (particularly education) which may contribute to this difference. However, across all communities, more F3HK participants had finished college than was typical in their communities, even in NC2 where a college degree was rare. Three prior CO-CSA studies in rural and micropolitan areas also noted high levels of education among participants^(17,18,25)^. This suggests that CO-CSA programmes may have more difficulty reaching residents with lesser education either due to recruitment approaches or because programme operations do not meet their needs. Prior research has documented low levels of awareness of CSA among both urban^(36)^ and rural^(37)^ residents with low incomes. Since awareness of and knowledge about CSA is a necessary precursor to participation^(38)^, a lack of familiarity with CSA may inhibit enrolment. Extension educators could conduct community-wide CSA awareness activities to support reach among those with fewer resources. Future research should explore methods for improving reach of CO-CSA to residents with less formal education.

All F3HK participants had incomes at 185 % Federal Poverty Level or below, but participants who had relatively more education or other resources (higher income, and spouse or other adult living in the household) picked-up CO-CSA shares a greater percentage of weeks than their counterparts with fewer resources. Married participants also attended education sessions more often. This suggests that, even if successfully recruited to a CO-CSA, participants from low-income households may need both a cost-offset and relatively more household resources to fully participate. Conversely, CO-CSA programmes may need to provide a cost-offset and other resources or supports to facilitate full participation. F3HK provided nutrition education and cooking tools as additional supports, but the results presented here suggest that more foundational resources like money and time were needed for some participants to fully participate. Future research is needed to disentangle the effects of these inter-related resources on participation and to explore approaches to meeting these needs.

Our data also illustrate that attendance at education sessions was consistently lower than CO-CSA pick up participation, and in five communities was 40–60 percentage points lower. This suggests that local context may matter differently for pick up and attendance, and programme design may require different adaptations for CO-CSA and for education. Future CO-CSA programmes should aim to identify local factors that could potentially hinder participation levels (rural location, low education, lower income, and no other adults in the household), and address these barriers through local programme adaptation. Research is needed to better understand how to encourage increased CO-CSA participation levels among participants with lower socioeconomic status, so as not to exacerbate existing health disparities.

### CO-CSA operational practices that may support participation

This study identified offering multiple share sizes and a community pick up location as operational practices associated with CO-CSA participation levels. Formative research for F3HK had suggested that potential CO-CSA participants desire control over the variety and quality of produce provided through mechanisms such as multiple share sizes and market-style selection^(37)^, as well as convenient pick up location^(37,39,40)^. Most F3HK partner farms offered flexibility in share size, pick up location or both, which is consistent with the operational characteristics of CO-CSA programmes in prior studies which frequently offered multiple share sizes^(14,16–18,25)^ and multiple pick up locations^(16–18,23,25)^. Farms that offered multiple share sizes generally had participants who both selected the larger share, and had higher levels of CO-CSA pick up, suggesting both positive attitudes toward FV and the logistical capacity for consistent pick up among this subset of participants.

When offered a choice of pick up locations, participants often selected a convenient community site. Prior research also suggests that convenience as perceived by participants also may include a familiar socio-demographic environment in which they feel welcome^(40)^, which many community locations provided (e.g. housing complex, community church). However, participants at community pick up sites also had *lower* levels of CO-CSA pick up than at other types of locations. For example, in WA1, participants lived in the housing complex that served as the setting for both CO-CSA pick up and classes. Yet despite the convenience of this community location, 9 (43 %) participants attended no classes and 7 (33 %) participants missed pick ups (most of whom eventually dropped out). Together these findings suggest that participants who prefer community pick up locations also may have additional barriers to participation. NC2 operated solely with a community pick up site and the overall poor participation in this location may have influenced these results.

Some prior studies reported on participants’ satisfaction with cost^(14,16–18,25)^, pick up location^(14,16–18,25,40)^ and share volume^(14,16–18,25)^. However, no prior study that reported on participation level^(14,17,19,22)^ also tested associations between programme operations and participation levels. Our study, therefore, provides novel evidence on associations between the choice of share size and a community pick up location with participation levels. In addition, prior studies suggest that CO-CSA farms that offer some flexibility to participants may be more effective than more rigid programmes. Three CO-CSA effectiveness studies offered little flexibility to participants and all reported no change in FV intake^(14,20,22)^. CO-CSA studies that included flexibility to participants by offering a few options^(15,19,21)^, many options^(17)^ or options that varied because they partnered with multiple farms^(16,18,23,25)^, tended to report positive outcomes.

Assessing and understanding local residents’ needs and desires related to CO-CSA operational practices (especially choice of share size and community pick up location) prior to programme implementation could support the local tailoring of operations to match these needs and, thus, may support participation. Future research should test associations between CO-CSA operational practices and CO-CSA effectiveness using rigorous research design and larger samples.

### Limitations

Several limitations of this study deserve note. First, the sample sizes are small which limits statistical power. Although the F3HK intervention enrolled a total of 148 caregiver–child dyads into the intervention group, the contexts for implementation resulted in small local samples (two of which had fewer than eight participants and were excluded from analyses). In particular, NC2 emerged as unique as having the lowest education and income, and was also the only community classified as entirely rural according to RUCA codes^(32)^. Second, selection of communities was primarily guided by the availability of both an interested partner farm and an available local extension educator. This was a difficult pairing to identify and was most difficult in NC where one community slightly exceeded population criteria and no appropriate and willing extension educator could be identified and therefore staff provided the CSA-tailored nutrition education lessons. The inclusion of only one entirely rural town and this selection process together may hinder the generalisability of these results to other locations. Third, although the F3HK intervention trial required partner farms to accept both weekly payments and to include SNAP as an accepted form of payment, other operational characteristics were decided upon and implemented by partner farms. Although acceptance of SNAP benefits was required, only 24 % of participants used SNAP benefits all or most weeks^(28)^. Farms likely select their practices in consideration of the local community context which limits our ability to disentangle associations among contextual characteristics, CO-CSA operational practices and participation levels.

## Conclusion

Small towns and micropolitan communities are highly varied in their population characteristics, and CO-CSA does not appear equally well-suited to all implementation contexts. Understanding community characteristics and familiarity with CSA and adapting models to address potential participation barriers such as limited education and financial resources are important to local CO-CSA programme adaptation. Flexibility in CO-CSA operational practices, and particularly offering multiple share sizes, may support recruitment and participation levels. However, although community pick up locations are desired by participants, these enrollees may face challenges to participation. Future research is needed to better understand barriers to participation in CO-CSA, particularly in rural communities and among participants utilising community pick up locations.
